# Beyond *p*-values: a cross-sectional umbrella review of chemotherapy-induced peripheral neuropathy treatments

**DOI:** 10.3389/fpain.2025.1564662

**Published:** 2025-03-19

**Authors:** Alice L. Ye, Salahadin Abdi

**Affiliations:** Department of Pain Medicine, The University of Texas MD Anderson Texas Center, Houston, TX, United States

**Keywords:** chemotherapy-induced peripheral neuropathy, effect size analysis, umbrella review, systematic reviews, randomized controlled trials, clinical research methodology, *p*-values

## Abstract

**Introduction:**

Chemotherapy-induced peripheral neuropathy (CIPN) is a common side effect of neurotoxic chemotherapy agents, significantly impacting the daily lives of many cancer survivors. Despite thousands of articles published on CIPN, we remain no closer to a successful treatment regimen for the condition. In recent years, several new clinical trials and systematic reviews have been published, many exploring nonpharmaceutical interventions, prompting the need for a comprehensive synthesis of this emerging evidence.

**Methods:**

We conducted an umbrella review to identify and appraise the 19 systematic reviews (SRs) published in 2023 that examined randomized controlled trials (RCTs) for established CIPN treatment. We focused our analysis on the three most researched treatment options: oral drugs, exercise, and acupuncture. RCTs not previously synthesized together were reviewed, and effect size analyses were performed to allow readers to interpret the existing literature beyond binary *p*-values.

**Results:**

Our analysis of RCTs revealed the following key findings. For cancer survivors with CIPN after completing chemotherapy, serotonin-norepinephrine reuptake inhibitors (SNRIs) as well as acupuncture provided at least short-term relief for pain and sensory symptoms. For patients with CIPN who were actively undergoing chemotherapy, home-based balance and strength training exercises appeared to alleviate symptoms. Effect size analyses highlighted variability in treatment responses, underscoring the limitations of relying solely on *p*-values to assess intervention efficacy.

**Discussion:**

Through an umbrella review approach, we demonstrate that SRs are often less systematic than expected. None of the 19 SRs captured all relevant RCTs within their search timeframe. However, by cross-referencing SRs, we identified 41 RCTs across 42 publications, illustrating the feasibility of an umbrella review approach to uncover relevant trials. Furthermore, many SRs exhibited methodological concerns that limit the interpretability of their findings. Finally, we discuss multiple opportunities for refining methods and reporting in future CIPN treatment trials.

**Systematic Review Registration:**

https://www.crd.york.ac.uk/PROSPERO/view/CRD42024508283, PROSPERO (42024508283).

## 1 Introduction

Chemotherapy-induced peripheral neuropathy (CIPN) remains a difficult-to-treat consequence of neurotoxic chemotherapy agents. It commonly presents as a painful sensory neuropathy in 68% of patients within the first month of chemotherapy and persists as a chronic condition in up to 30% of patients (1). As both global demands for chemotherapy and the rate of cancer survivorship increase, it is expected that an increasing number of patients will develop and live with CIPN (2, 3). Unfortunately, there are severely limited treatment options for CIPN despite the abundance of published literature. Since the release of the 2020 American Society for Clinical Oncology (ASCO) CIPN guidelines—which stated that duloxetine is the only intervention with evidence for treating CIPN—there have been several new clinical trials published, many exploring nonpharmaceutical interventions (4). The number has been enough to stimulate the publication of 19 systematic reviews (SRs) on CIPN treatment in the year 2023 alone (5–24). However, the findings from these SRs often have conflicting conclusions. For example, while the D'Souza 2023 review agrees with the 2020 ASCO guidelines on duloxetine, the Chow 2023 review concludes that duloxetine appears to be similar to placebo (7, 10). Ultimately, the heterogeneity of these reviews frequently leads to conflicting conclusions, hindering the development of standardized treatment strategies.

We demonstrate that individual SRs, even with thorough search strategies, struggle to comprehensively identify all relevant randomized controlled trials (RCTs). However, when pooled together, a search across these 19 SRs identifies 41 unique RCTs (42 total articles) not previously analyzed together. We focus here on the most researched treatment options for improving pain and sensory outcomes in patients with CIPN, summarizing the evidence from 22 RCTs to better estimate the true efficacy of pharmaceutical agents like duloxetine, exercise therapy, and acupuncture interventions. For each intervention category, we present relevant outcomes across trials, discuss trial contributions and pitfalls, and provide effect size analyses on each intervention's overall clinical utility for those with CIPN. By emphasizing effect size analysis over binary statistical significance, this review focuses on the magnitude of treatment effects rather than solely on *p*-values, aiming to provide a more nuanced perspective on treatment efficacy. Lastly, we will review emerging treatment options on the horizon for CIPN.

Furthermore, we discuss critical challenges in CIPN research, such as the need for improved clinical trial designs, refined outcome measures, and enhanced data reporting practices. These methodological advancements are essential for addressing the current limitations in the field and for fostering meaningful progress in CIPN treatment development. By broadening the scope of evidence synthesis and highlighting key research gaps, this review seeks to support the scientific community in advancing our understanding and management of CIPN.

## 2 Methods

The protocol was registered with PROSPERO (42024508283). The original protocol outlines a broader SR across multiple years. For this article, we narrowed the scope to focus specifically on the 19 SRs published in 2023 to synthesize outcomes from the most researched interventions. This focused approach was chosen to facilitate a timely effect size analysis of the reported outcome data, offering a clearer understanding of treatment efficacy and addressing inconsistencies in the existing literature for the most common interventions. As the focus of this review is on synthesizing effect sizes from the RCTs identified across SRs, no risk of bias assessment of the systematic reviews themselves are presented here. Otherwise, the review adhered to the guidelines outlined in the Preferred Reporting Items for Systematic Reviews and Meta-Analyses (PRISMA).

Systematic reviews were included if they were published in the English language in 2023, included RCTs for established CIPN, including both pharmaceutical and nonpharmaceutical interventions, and evaluated CIPN-related outcomes, including pain, sensory symptoms, or functional impairment. SRs were excluded if they focused on preventative strategies for CIPN and if they did not directly cite RCTs in their bibliographies and thus introduced greater risk of unverifiable data. A comprehensive search was conducted across the MEDLINE, Embase, and Cochrane Library databases to identify relevant systematic reviews published between January 1, 2023, and December 31, 2023, with an updated search on August 8, 2024 of the 2023 timeframe to ensure that any delayed publications were captured. The search strategy was developed in collaboration with a medical librarian and included the terms: “peripheral neuropathy”, “systematic review”, “cancer”. The full search strategy is available in the Supplemental Materials.

For the effect size analysis of RCTs, we defined RCTs as having either passive control arms (usual care or waitlist control) or active control arms (education, sham, or placebos). We excluded trials that were comparative between two experimental arms such as trials comparing endurance vs. strength training. Calculations of primary data, when available, follow the formulas recommended in the 2023 Cochrane Handbook for Systematic Reviews and worst-case scenarios are used when calculating relative risks (RRs): missing data in the intervention arm is counted toward negative outcomes while missing data in the control arm is counted toward positive outcomes (25).

To our knowledge, this is the first review to categorize participants in CIPN treatment trials based on chemotherapy status, distinguishing between those undergoing chemotherapy concomitantly (ongoing neurotoxic insult) and those participating in the intervention after completing chemotherapy (no active neurotoxic insult). This distinction may be clinically significant, as certain interventions might be more effective when administered during active neurotoxicity, while others more effective after the injury process has stabilized.

Two independent reviewers (ALY, AB) screened the identified SRs for eligibility, with discrepancies resolved through discussion with a third reviewer (SA). A single reviewer (ALY) extracted RCT data from the final selection of SRs. Extracted data included key characteristics of the RCTs, such as outcome measures, interventions, sample size, *p*-values, means, measures of variability, and confidence intervals. A meta-analysis was not performed to maintain the focus on effect size results and on trial-specific details.

## 3 Results

**Figure 1:**
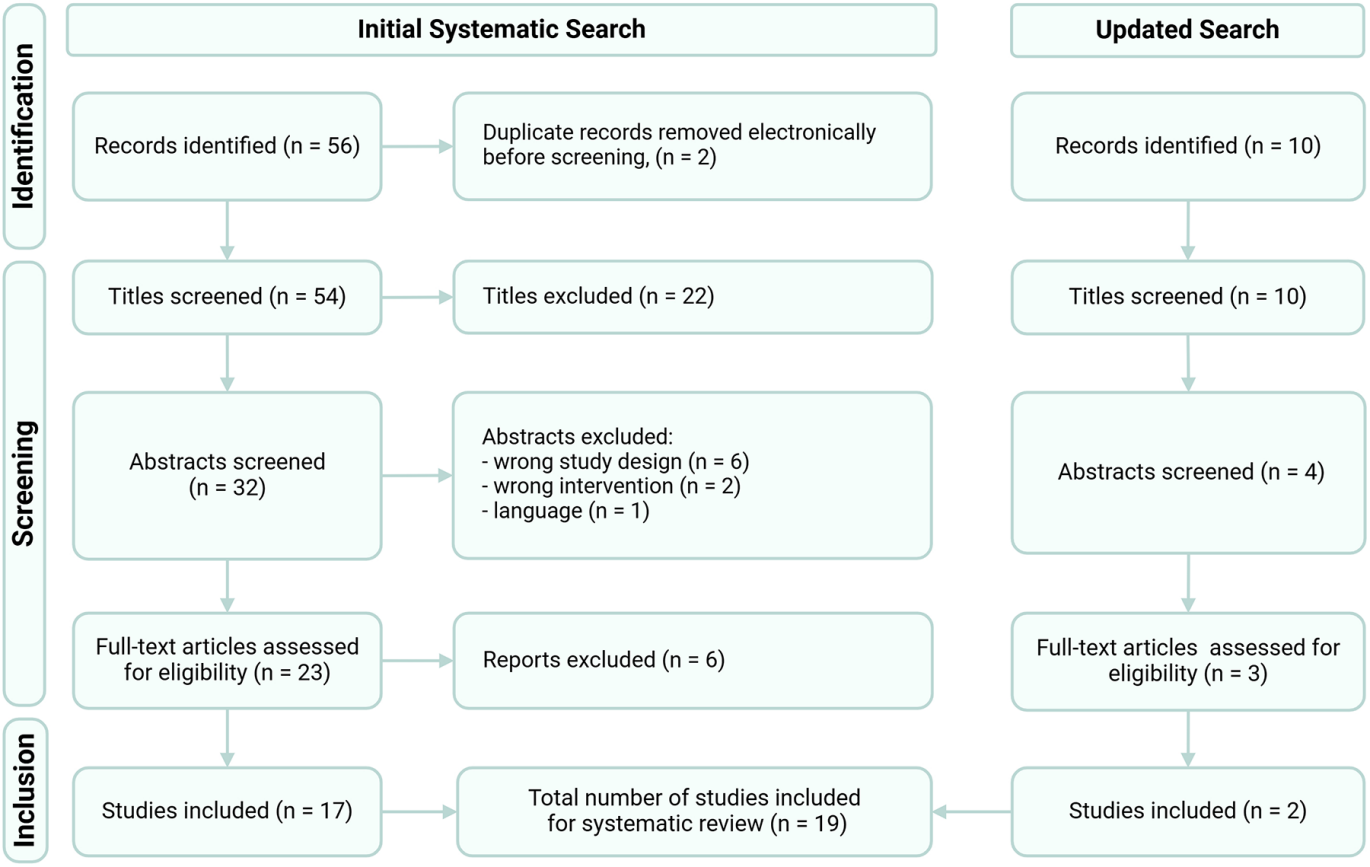
PRISMA flow diagram of umbrella review study selection.

In 2023, 19 SRs on RCT interventions for established CIPN were identified (Figure 1) (5–21, 23, 24). Across these SRs, a total of 41 unique RCTs (spanning 42 publications) were found, including 13 RCTs published between 2021 and 2023, after the release of the 2020 ASCO guidelines. Notably, many individual SRs demonstrated limitations in systematically identifying all relevant RCTs. For example, 32% of SRs reported RCTs that were not captured by other reviews on similar topics and the median number of RCTs identified per review was only 2 (Table 1). Additionally, 79% of SRs included comparative trials in their synthesis (i.e., comparing two experimental arms) and 63% combined evidence from prevention (patients without CIPN) and treatment (patients with established CIPN) trials, creating challenges in interpreting results.

**Table 1:** Overview of 2023 published systematic reviews for chemotherapy-induced peripheral neuropathy treatment.

Feature	Count (%)
Reviews that included comparative trials	15/19 (79%)
Reviews that combined preventative and treatment trials	12/19 (63%)
Reviews with limited search timeframes	1/19 (5%)
Reviews that provided *only p*-values of trials	3/19 (16%)
Reviews that provided effect sizes of trials	15/19 (79%)
Reviews that had an RCT not reported in other reviews	6/19 (32%)
Number of RCTs identified per review, median (range)	2 (1–23)

RCT, randomized controlled trial.

Even the most comprehensive reviews had notable gaps. For instance, D'Souza 2023 identified all pharmaceutical RCTs but missed three exercise- and acupuncture-related RCTs within their search timeframe (10). Zhang 2023 identified all acupuncture related RCTs but omitted several exercise trials (24). Despite employing thorough search strategies, individual SRs were prone to missing relevant RCTs, reinforcing the value of pooling results across multiple SRs. This umbrella review approach enabled a more exhaustive identification of RCTs, uncovering a wider range of interventions, including RCTs for the most common interventions: oral drugs (6 RCTs), exercise (8 RCTs), and acupuncture (8 RCTs across 9 publications) (26–48).

The remaining RCTs encompassed less studied interventions such as touch therapies (Reiki, reflexology, acupressure) (47, 49–53), behavioral therapies (54–56), subcutaneous injections (57), topical agents (58–60), mucosal sprays (61), supplements (29, 62), cold therapy (43), meditation (47), hyperbaric oxygen (29), and neuromodulatory treatments (photobiomodulation and electrical stimulation) (63–67). Outcomes from these experimental interventions are more thoroughly analyzed in a pending manuscript.

Tables 2–4 present the effect size analyses of 22 RCTs involving oral drug, exercise, and acupuncture interventions, far more than the median of 2 RCTs that we found were identified per individual SR. Of these 22 RCTs, only 7 were discussed in the 2020 ASCO guidelines and 6 were published after the guidelines were released. By focusing on effect sizes rather than binary statistical significance via *p*-values, we sought to provide nuanced insights into treatment efficacy. These tables offer comparison statistics and detailed background information, such as patient population characteristics, sample sizes, outcome measures, and time points, enabling researchers to draw their own conclusions.

**Table 2:** Symptom outcomes from randomized controlled trials for oral drugs.

Treatment, duration (analyzed *n*)	Timepoint	Outcome Measure (range)^a^	MD/RR (95% CIs)
Serotonin-Norepinephrine Reuptake Inhibitors (2 studies) (36, 37)			
Farshchian 2018, 4 weeks, after chemotherapy			
Duloxetine 30 mg/days (52) vs. placebo (52)	EOT	Having mild or no sensory neuropathy per NCI-CTCAE v2^b^	*4.09 RR (2.39, 6.99), NNT 2* *
	EOT	Having mild or no neuropathic pain per NCI-CTCAE v2	*2.22 RR (1.63, 3.02), NNT 2* *
Smith 2013, 5 weeks, after chemotherapy			
Duloxetine 60 mg/days (87) vs. placebo (94), crossover trial with only 1st arm analyzed.	EOT	≥50% pain reduction	2.43 RR (1.11, 5.30), NNT 9*
	EOT	Pain (0–10 W), average	-0.73 MD (−1.20, −0.26)*
	EOT	BPI Pain Interference (0–70 W)	−4.40 MD (−7.88, −0.93)*
	EOT	FACT/GOG-Ntx (W0–44)	1.58 MD (0.15, 3.00)*
Farshchian 2018, 4 weeks, after chemotherapy			
Venlafaxine 37.5 mg/days (52) vs. placebo (52)	EOT	Having mild or no sensory neuropathy per NCI-CTCAE v2	*3.55 RR (2.05, 6.13), NNT 2* *
	EOT	Having mild or no neuropathic pain per NCI-CTCAE v2	*1.70 RR (1.20, 2.39), NNT 4* *
Gabapentinoids (2 studies) (38, 40)			
Rao 2007, 6 weeks, undergoing (50%) or after chemotherapy (50%)			
Gabapentin 300 mg/days to 2,700 mg/days (57) vs. placebo capsules (58), crossover trial with only 1st arm analyzed.	EOT	Pain (0–10 W), average	*−0.1 MD (−0.29, 0.09)*
	EOT	WHO Neuropathy Scale (0–4 W)	*0.1 MD (−0.41, 0.61)*
Hincker 2019, 4 weeks, after chemotherapy			
Pregabalin 75 mg BID to 300 mg BID (25) vs. placebo capsules (25), crossover trial with both arms analyzed pairwise.	EOT	≥50% average pain reduction	*1.67* *RR (0.45, 6.24)*
	EOT	≥50% worst pain reduction	*1.75* *RR (0.58, 5.24)*
	EOT	Pain (0–10 W), average	*0.1* *MD (−1.04, 1.24)*
	EOT	Pain (0–10 W), worst	*−0.2* *MD (−1.48, 1.08)*
Tricyclic Antidepressants (2 studies) (35, 39)			
Kautio 2009, 8 weeks, undergoing chemotherapy			
Amitriptyline 10 mg to 50 mg/days (17) vs. placebo pills (16)	EOT	Global improvement of neuropathic symptoms (W0–4)	*1.5* *MD (−1.29, 4.29)*

	EOT	At least some relief of neuropathic symptoms	*1.51 RR (0.62, 3.65)*
	EOT	Neuropathic pain scale (NA)	*p-value >0.05 assumed*
Hammack 2002, 4 weeks, undergoing (14%) or after chemotherapy (86%)			
Nortriptyline 25 mg to 100 mg/days (26) vs. placebo tablets (25), crossover trial with only 1st arm analyzed.	EOT	Pain or tingling (0–100 W)	*−5.0 MD (−39.89, 29.89)*
	EOT	Pain or tingling (0–4 W)	*−0.1 MD (−15.66, 15.46)*

Italicized values are calculated from the data available, assuming worst-case scenarios for outcomes in case of missing binary data. Sample size numbers may differ for different outcomes due to missing data. MD, mean difference of intervention minus control; RR, relative risk (given whenever possible); CI, confidence intervals; NNT, number needed to treat; EOT, end of treatment period; BPI, Brief Pain Inventory (pain inference subscale measures functional limitations from pain); FACT/GOG-Ntx, Functional Assessment of Cancer Therapy/Gynecologic Oncology Group-Neurotoxicity (measures neuropathic symptoms); NCI-CTCAE, National Cancer Institute-Common Toxicity Criteria for Adverse Events (physician-rated grading system for symptoms); WHO, World Health Organization (WHO Neuropathy Scale measures symptom severity); BID, *bis in die*, for twice daily; NA, not available.

^a^ W indicates which end of the outcome scale is worse.

^b^ NCI-CTCAE measure assumed based on results.

* *p* < 0.05.

**Table 3:** Symptom outcomes from randomized controlled trials for exercise.

Treatment, duration (analyzed *n*)	Timepoint	Outcome Measure (range)^a^	MD/RR (95% CIs)
Yoga (3 studies) (45–47)			
Clark 2012, 6 weeks, after chemotherapy			
Yoga weekly (7) vs. neuropathy education (7)	EOT	FACT/GOG-Ntx (W0–44)	*4.57 MD (−4.03, 13.17)*
Bao 2020b, 8 weeks, after chemotherapy			
Yoga daily (21) vs. usual care (20)	EOT	FACT/GOG-Ntx (W0–44)	2.88 MD (0.20, 5.56)*
	EOT	Numbness (0–10 W)	0.39 MD (−0.97, 1.75)
	EOT	Tingling (0–1 0W)	−0.25 MD (−1.67, 1.17)
	EOT	Pain (0–10 W)	−1.30 MD (−3.01, 0.41)
	FU, 4 weeks	FACT/GOG-Ntx (W0–44)	4.03 MD (1.38, 6.68)*
	FU, 4 weeks	Pain (0–10 W)	−1.94 MD (−3.65, −0.24)*
Knoerl 2022, 8 weeks, after chemotherapy			
Yoga formal program (23) vs. usual care (14)	EOT	Pain (0–10 W), worst	0.74 *p*-value
	EOT	EORTC QLQ-CIPN20, Sensory Subscale (0–100 W)	0.42 *p*-value
Balance Training Only (2 studies) (41, 48)			
Schwenk 2016, 4 weeks (8 sessions), after chemotherapy			
Sensor feedback-based balance training (11) vs. non-specific exercise encouragement (11)	EOT	FES-I (16–64 W)	*1.5* *MD (−1.30, 4.30)*
Streckmann 2018, 6 weeks (12 sessions), after chemotherapy			
Progressive balance training (10) vs. no intervention control (10)	EOT	FACT/GOG-Ntx (W0–44)	0.096 *p*-value
	EOT	Pain-DETECT (3 categories)	*data NA*
Balance and Strength Training (2 studies) (43, 44)			
Dhawan 2020, 10 weeks (daily), undergoing chemotherapy			
Home strengthening and balance exercises (19) vs. usual care (22)	EOT	Reporting no tingling or prickling of skin	*3.14 RR (0.35, 27.92)*
	EOT	LANSS (2 categories)	*binary data NA*
	EOT	CIPNAT Symptoms (0–279 W)	*−57.7 MD (−84.07, −31.33)* *
	EOT	CIPNAT Interference (0–140 W)	*−22.0* MD (−32.06, −11.94)*
Simsek and Demir 2021, 12 weeks (5x/week), undergoing chemotherapy			
Home strengthening, balance, and stretching exercises (30) vs. usual care (30), hands and feet outcomes are averaged together	EOT	Pain (0–10 W)	*−2.9* MD (−3.87, −1.93)*
	EOT	Numbness (0–10 W)	*−1.2* MD (−2.17, −0.23)*
	EOT	Tingling (0–10 W)	*−1.6* MD (−2.61, −0.59)*
Hands Only Training (1 study) (42)			
Ikio 2022, 6–8 weeks (3x/week), undergoing chemotherapy			
Unsupervised hand strength, sensory, and dexterity training with usual care (15) vs. usual care alone (14)	EOT	Hand Pain (0–100 W)	−4.57 MD (−14.95, 5.81)
	EOT	Neuropathic Symptoms (0–100 W)	−6.27 MD (−15.68, 3.14)

Italicized values are calculated from the data available, assuming worst-case scenarios for outcomes in case of missing data. Sample size numbers may differ for different outcomes due to missing data. MD, mean difference of intervention minus control; RR, relative risk (given whenever possible); CI, confidence intervals; NNT, number needed to treat; EOT, end of treatment period; FACT/GOG-Ntx, Functional Assessment of Cancer Therapy/Gynecologic Oncology Group-Neurotoxicity (measures neuropathic symptoms); EORTC QLQ-CIPN20, European Organization for Research and Treatment of Cancer Quality of Life Questionnaire Chemotherapy-Induced Peripheral Neuropathy 20 (measures symptoms and functional limitations); FES-I, Falls Efficacy Scale-International (measures fear of falling); Pain-DETECT, screening measure for 3 pain categories (nociceptive, unclear, or neuropathic); LANSS, Leeds Assessment of Neuropathic Symptoms and Signs (screening measure for unlikely vs. likely neuropathic pain); CIPNAT, Chemotherapy-induced Peripheral Neuropathy Assessment Tool with 2 subscales (symptoms and interference).

^a^ W indicates which end of the outcome scale is worse.

* *p* < 0.05.

**Table 4:** Symptom outcomes from randomized controlled trials for acupuncture.

Treatment, duration (analyzed *n*)	Timepoint	Outcome Measure (range)^a^	MD/RR (95% CIs)
Needle acupuncture only (5 studies) (30–34)			
Molassiotis 2019, 8 weeks (16 sessions), undergoing (10%) or after chemotherapy (90%)			
Needle acupuncture (44) vs. usual care (43)	EOT	FACT/GOG-Ntx (W0–44)	*10 MD (2.47, 17.53)* *
	EOT	Having mild or no sensory neuropathy per NCI-CTCAE v4	*1.61 RR (1.05, 2.48), NNT 5* *
Stringer 2022, 10 weeks (10 sessions), undergoing (58%) or after chemotherapy (42%)			
Needle acupuncture with usual care (61) vs. usual care only (59)	EOT	Having mild or no sensory neuropathy per NCI-CTCAE v4	*3.73 RR (1.76, 7.90), NNT 4* *
	EOT	Having ≥2 point improvement in MYMOP (0–6 W)	*1.58 RR (1.07, 2.34), NNT 5* *
	EOT	EORTC QLQ-CIPN20 (0–100 W)	−12.66 MD (−18.11, −7.21)*
D'Alessandro 2019, 5 weeks (10 sessions), after chemotherapy			
Needle acupuncture with rehabilitation (12) vs. usual neuropathy rehabilitation alone (9)	EOT	Pain (0–10 W)	*−0.60 MD (−3.15, 1.95)*
Molassiotis 2019, 8 weeks (16 sessions)			
Needle acupuncture (44) vs. usual care (43)	EOT	Pain (0–10 W), worst	*−0.70 MD (−1.69, 0.29)*
Huang 2021, 9 weeks (15 sessions), after chemotherapy			
Needle acupuncture (10) vs. sham shallow needling (10)	EOT	Pain (0–10 W), average	*−1.82 MD (−3.42, −0.22)* *
Stringer 2022, 10 weeks (10 sessions)			
Needle acupuncture with usual care (53) vs. usual care only (55)	EOT	Pain (0–10 W), worst	−1.61 MD (−2.39, −0.83)*
Friedemann 2022, 10 weeks (10 sessions), after chemotherapy			
Needle acupuncture (51) vs. waitlist (51), crossover trial with both arms analyzed.	FU, 4 weeks	Pain (0–10 W), neuropathic and burning	*−1.29 MD (−2.18, −0.39)* *
	FU, 4 weeks	Numbness (0–10 W)	*−1.56 MD (−2.26, −0.66)* *
	FU, 4 weeks	Tingling (0–10 W)	*−0.83 MD (−1.92, 0.26)*
Needle acupuncture with electrical stimulation (4 studies) (26–29)			
Rostock 2013, 3 weeks (7–9 sessions), after chemotherapy			
Electroacupuncture (14) vs. placebo pill (17)	EOT	Having mild or no sensory neuropathy per NCI-CTCAE v2	*0.95 RR (0.67, 1.36)*
	EOT	Neuropathy symptoms (0–10 W)	0.3 MD (−0.8, 1.4)
Lu 2020, 8 weeks (18 sessions), after chemotherapy			
Electroacupuncture (16) vs. usual care (17), crossover trial with only 1st arm analyzed.	EOT	PNQ Sensory Scale (0–4 W)	*−0.70 MD (−1.24, −0.16)* *
	EOT	FACT/GOG-Ntx (W0–44)	*7.5 MD (2.31, 12.69)* *
Bao 2020a, Bao 2021, 8 weeks (10 sessions), after chemotherapy			
Electroacupuncture (24) vs. usual care (21)	EOT	FACT/GOG-Ntx (W0–44)	4.17 MD (1.62, 6.72)*
	FU, 4 weeks	FACT/GOG-Ntx (W0–44)	1.86 MD (−0.68, 4.41)
Lu 2020, 8 weeks (18 sessions), after chemotherapy			
Electroacupuncture (14) vs. usual care (17)	EOT	Pain (0–10 W), average	*−1.70 MD (−2.95, −0.45)* *
Bao 2020a, Bao 2021, 8 weeks (10 sessions), after chemotherapy			
Electroacupuncture (24) vs. usual care (21)	EOT	Pain (0–10 W)	*−1.56* MD (−2.93, −0.19)*
	EOT	Numbness (0–10 W)	*−2.11* MD (−3.59, −0.63)*
	EOT	Tingling (0–10 W)	*−1.69* MD (−3.23, −0.15)*
Electroacupuncture (24) vs. non-invasive sham acupuncture (23)	EOT	FACT/GOG-Ntx (W0–44)	−0.77 MD (−3.25, 1.71)
	EOT	Pain (0–10 W)	*−0.84 MD (−2.28, 0.60)*
	EOT	Numbness (0–10 W)	*−0.02 MD (−1.40, 1.36)*
	EOT	Tingling (0–10 W)	*−0.61 MD (−2.01, 0.79)*

Italicized values are calculated from the data available, assuming worst-case scenarios for outcomes in case of missing data. Sample size numbers may differ for different outcomes due to missing data. MD, mean difference of intervention minus control; RR, relative risk (given whenever possible); CI, confidence intervals; NNT, number needed to treat; EOT, end of treatment period; FACT/GOG-Ntx, Functional Assessment of Cancer Therapy/Gynecologic Oncology Group-Neurotoxicity (measures neuropathic symptoms); NCI-CTCAE, National Cancer Institute-Common Toxicity Criteria for Adverse Events (physician-rated grading system for symptoms); MYMOP, Measure Yourself Medical Outcome Profile (patient-individualized grading system for their most significant symptoms); EORTC QLQ-CIPN20, European Organization for Research and Treatment of Cancer Quality of Life Questionnaire Chemotherapy-Induced Peripheral Neuropathy 20 (measures symptoms and functional limitations).

^a^ W indicates which end of the outcome scale is worse.

* *p* < 0.05.

### 3.1 Oral drugs

Anticonvulsants (gabapentin, pregabalin) and antidepressant medications [serotonin-norepinephrine reuptake inhibitors (SNRIs) and tricyclic antidepressants (TCAs)] are often prescribed for peripheral neuropathy conditions like diabetic neuropathy (68, 69). It is thought that these oral drugs work through downstream peripheral and central mechanisms to transiently inhibit pain processing pathways, thus requiring consistent use for continued pain relief (70). We identified 6 RCTs cited across SRs that compared placebo pills against gabapentin (38), pregabalin (40), duloxetine (36, 37), venlafaxine (36), amitriptyline (35), and nortriptyline (39). Table 2 summarizes symptom outcomes from these trials as either the mean difference (MD) between intervention and placebo or as the relative risk/benefit (RR) of the intervention, with 95% confidence intervals (CIs).

#### 3.1.1 Duloxetine and venlafaxine

Duloxetine and venlafaxine are well-known SNRIs used for mood disorders and certain neuropathic pain conditions. Duloxetine is the only agent recommended for treating painful CIPN by the 2020 ASCO Guidelines, a recommendation that was reaffirmed in the D'Souza 2023 review but contradicted in the Chow 2023 review (4, 10). Both D'Souza 2023 and Chow 2023 assess results from the Smith 2013 and Farshchian 2018 RCTs (Table 2) (7, 36, 37).

Smith 2013 investigated duloxetine 60 mg daily vs. placebo in patients with CIPN following taxane or oxaliplatin treatment in a crossover trial design. The primary endpoint was set at the end of the 1st crossover period, with 181 patients analyzed. Although pairwise analysis of both crossover periods could have improved the study's power, the results from the initial period were promising. After 5 weeks, the initial duloxetine group reported an average pain intensity reduction of 0.73 (on a 0–10 scale) compared to placebo (Table 2). The RR of duloxetine for patients self-reporting at least 50% pain reduction was 2.42 RR (1.11–5.30, 95% CI), with a number needed to treat (NNT) of 9. This means that 9 individuals would need to receive duloxetine for 1 individual to achieve at least 50% pain reduction. Additional benefits were seen for improvements in pain interference and neuropathic symptoms (71). Subgroup analysis suggested differential responses based on chemotherapy class, with taxane-treated patients potentially less responsive than those treated with oxaliplatin. These findings warrant further studies to confirm chemotherapy-specific responses with duloxetine. The dropout rate in the duloxetine arm (19%, 21/109) exceeded that of the placebo group (11%, 12/105), though this difference was not statistically significant (*p* = 0.13, Fisher's Exact test).

Farshchian 2018 later conducted a 4-week three-arm trial comparing duloxetine 30 mg daily, venlafaxine 37.5 mg daily, and placebo (52 participants in each arm, *N* = 156) for five well-distributed chemotherapy protocols (*p* = 0.95) (36). Despite its shorter duration and lower duloxetine dosage, the trial showed that more participants in the duloxetine arm achieved mild or no sensory neuropathy status compared to placebo, with an RR of 4.09 (95% CI: 2.39–6.99) and an NNT of 2. Similarly, duloxetine significantly improved neuropathic pain, with an RR of 2.22 (95% CI: 1.63–3.02) and an NNT of also 2. Venlafaxine also outperformed placebo, with an RR of 3.55 (95% CI: 2.05–6.13) for sensory neuropathy (NNT = 2) and an RR of 1.70 (95% CI: 1.20–2.39) for neuropathic pain (NNT = 4). One possible interpretation of the different NNT values is that achieving a CTCAE rating of mild or no neuropathic pain may be easier than achieving ≥50% pain reduction on the NRS. However, this must be tempered with the knowledge that the CTCAE is observer-graded, with known issues of inter-rater variability, which may limit the precision of the estimated effect sizes (72, 73). This variability may make the NNT less reliable for CTCAE-based assessments. Thus, the results from Farshchian 2018 likely provides us with a general direction of treatment effects but may not be so useful in providing an accurate effect size. The conflicting results in the Chow 2023 review may stem from several methodological concerns, such as the inclusion of a comparative trial (duloxetine vs. vitamin B12) (74), inconsistent reporting of RR metrics (e.g., the inverse RR of an event occurring to the RR of an event not occurring are non-equivalent) (25), unreported cutoffs when transforming continuous outcomes into binary RRs, and pooling heterogeneous outcomes into an overall effect size.

In summary, our effect size analysis reinforces prior recommendations for duloxetine in patients with CIPN and provides additional evidence supporting venlafaxine, another SNRI, as a potential alternative. Notably, RCTs to date have only examined SNRIs in patients who have completed chemotherapy. For those who respond, benefits may appear within 4–5 weeks of therapy. However, the duration of these benefits remains uncertain, as does the potential efficacy of starting SNRIs during chemotherapy when CIPN symptoms may first appear. Future RCTs will need to be designed to target these questions, in addition to replicating and refining effect sizes.

#### 3.1.2 Other oral drugs

The 2020 ASCO guidelines concluded that no recommendations could be made regarding gabapentinoids or TCAs for treating CIPN, missing the mention of one relevant RCT on pregabalin from their search timeframe (40). D'Souza 2023 similarly concluded that studies are equivocal on gabapentinoids for CIPN, even when factoring in observational trials, and that TCAs do not reduce CIPN pain (10).

Based on 4 RCTs with placebo controls, Table 2 presents an analysis of non-SNRI oral drugs, showing that based on available data and incorporating data variance, gabapentin is unlikely to provide a meaningful difference as evidenced by its near-zero mean difference and tight CIs (*N* = 115). In contrast, pregabalin and TCAs may possibly offer small benefits, though this is conclusion is uncertain due to the wide CIs seen (*N* = 50 and 84, respectively). There were no RCTs found in SRs addressing opioids vs. placebo for CIPN treatment.

Additional high-quality research on pregabalin, TCAs, and opioids is needed but challenging. Because these RCTs may risk more harm than benefits, researchers may want to explore n-of-1 trial models. N-of-1 trials, also known as multiple crossover trials of single patients, help establish causality through multiple pairwise analyses when parallel-group RCTs may be inappropriate (75). High statistical power can be achieved with small sample sizes, fewer patients are exposed to potential risks, and aggregated n-of-1 trials can provide useful generalizable evidence.

### 3.2 Exercise therapy

Exercise has been shown to increase tissue regeneration across multiple organ systems including the peripheral nervous system, with strong preclinical evidence supporting its role in axonal regeneration and the amelioration of maladaptive nerve responses (76, 77). In 2023, nine SRs synthesized evidence of exercise therapies for CIPN regardless of cancer type or chemotherapy class (9, 10, 12, 16, 17, 19, 20, 23, 24). Two additional SRs focused specifically on exercise interventions in breast cancer patients (6, 15). Across all eleven SRs, eight exercise RCTs were identified, yet no single SR summarized the results from all eight trials together. SR conclusions to date have varied widely, from insufficient evidence to beneficial for pain but not other symptoms to beneficial for all CIPN symptoms. We present effect size analyses for all 8 RCTs in Table 3, dividing findings by yoga, balance training only, combined training, and hands-only training.

#### 3.2.1 Yoga

Three small RCTs examined yoga interventions compared to usual care or neuropathy education with sample sizes ranging from 14 to 37 participants. Data from Clark 2012 (*N* = 14) suggested a trend toward improved quality-of-life and/or symptom reduction with 6 weeks of weekly yoga sessions vs. education but had too wide CIs to draw conclusions, likely due to the small sample size (47). Bao 2020a (*N* = 41) reported modest benefits in symptom scores and a trend toward pain relief from 8 weeks of daily yoga vs. usual care (45). Interestingly, and with only minor drop-out, follow-up data collected 4 weeks post-intervention showed greater symptom relief and pain benefits for unclear reasons. More recently, Knoerl 2022 (*N* = 37) conducted a similar trial involving an 8-weeks formal yoga program vs. usual care showing non-significant changes in pain and sensory symptoms (46). Several design and reporting limitations make these findings difficult to interpret. The details of the yoga program were unclear, including how many sessions patients attended beyond the initial supervised session. Additionally, the presentation of median/range rather than mean/variance data hindered the replication of hypothesis testing and further assessment of whether their non-significant results are attributable to wide CIs.

Future research should prioritize targeted studies on post-intervention follow-up with improved methodological rigor to clarify whether yoga is helpful for CIPN symptoms. Unlike SNRIs, preclinical evidence suggests that exercise has regenerative properties, making follow-up data particularly important in yoga trials. For instance, if participants report continued benefits even after reducing or stopping yoga practice, this could indicate regenerative effects, as potentially observed in Bao 2020b. Future trials should also focus on accurately reporting on the dose and frequency of yoga and reporting standard mean/variance data to aid systematic reviews (78). Currently, there is insufficient evidence on whether yoga is beneficial for CIPN but management plans should consider whether yoga is appropriate to recommend for its well-documented psychosocial benefits in cancer patients (79).

#### 3.2.2 Balance training only

Two small RCTs evaluated balance-only training compared to non-specific exercise encouragement or unspecified control arms (Table 3) (41, 48). Schwenk 2016 (*N* = 22) tested wearable sensor devices in 8 balance training sessions over 4 weeks (41). At the end of treatment, participants reported on their fear of falling, with results showing a slight trend toward increased fear of falling in the balance training group compared to controls. However, the CI was too wide draw precise conclusions, as calculated using their adjusted *p*-value. Although baseline measures for numbness, tingling, or pain were collected, the study did not assess these symptoms at the end of treatment.

Streckmann 2018 (*N* = 20 of relevant arms) conducted 12 sessions of progressive balance training over 6 weeks (48). The study reported a *p*-value >0.05 for FACT/GOG-Ntx outcomes but did not provide effect sizes or CIs, making interpretation of the results challenging. Given the small sample sizes, non-significant *p*-values may reflect high variability within the sample rather than lack of treatment effect. Having effect size data and CIs would aid in answering whether this is the case. Their study also utilized Pain-DETECT, a neuropathic pain screening questionnaire, as a secondary outcome measure but did not report data comparing balance training vs. control (80).

Ultimately, small sample sizes and inadequate data reporting limit the ability to draw meaningful conclusions from these 2 RCTs on balance-only training for CIPN after chemotherapy completion. Additionally, no data are available on balance-only training for CIPN during ongoing chemotherapy. Future RCTs should consider including symptom outcome measures at time points beyond the baseline and endure reporting of treatment effects and variance alongside *p*-values.

#### 3.2.3 Balance and strength training

Two RCTs investigated near-daily to daily home-based balance and strength training vs. usual care for those with CIPN undergoing chemotherapy over 10–12 weeks (*N* = 41 and 60) (43, 44). In both RCTs, participants in exercise arms reported decreased severity of CIPN symptoms and functional interference compared to those receiving usual care (Table 3). Detailed training programs, available in the supplemental materials of both studies, include sitting, standing, and lying down exercises targeting the ankles, knees, hips, hands, and elbows. These findings suggest that yoga-based interventions, which feature incorporate balance and strength-based poses, might yield greater benefits if trial durations are extended for longer.

The benefit of one training program vs. the other cannot be directly compared because the two RCTs used different outcome measures. However, the results collectively indicate that consistent multimodal exercise over several weeks may benefit patients with CIPN undergoing chemotherapy.

Regarding trial quality, we draw attention to the use of the Leeds Assessment of Neuropathic Symptoms and Signs (LANSS) scale in Dhawan 2020, as highlighted in Table 3, to illustrate concerns about outcome measure selection (44). The LANSS scale, similar to the Pain-DETECT measure, is a neuropathic pain screening tool with questionable suitability for assessing CIPN (81). The scale was not designed for use in measuring symptom severity, and the relevance of individual questions within the scale for CIPN outcomes is unclear.

No follow-up data were collected in these studies, and there is no trial data on how multimodal exercise might benefit patients with CIPN after completing chemotherapy. Similar to the SNRI trials, it is unclear whether the observed benefits persist after the exercise is discontinued or significantly reduced. Additionally, while there is no evidence yet on the efficacy of multimodal exercise for patients with CIPN post-chemotherapy, exercise is a low-risk intervention with highly significant overall health benefits.

Future RCTs should address these gaps by designing trials with appropriate durations (10–12 weeks) with interim assessments and a follow-up periods. They should choose meaningful outcome measures, such as patient-reported symptoms severity and functional impact, and consider stratified randomization to ensure balanced sampling between CIPN patients undergoing chemotherapy and those who have completed chemotherapy. Additionally, future studies should investigate whether the benefits of multimodal exercise arise from specific exercises targeting neuropathy or increased physical activity in general.

#### 3.2.4 Hands-only training

One small RCT (*N* = 29) evaluated hands-specific training with usual care vs. usual care alone in patients with CIPN undergoing chemotherapy (42). Patients in the intervention arm followed an unsupervised hand-training protocol 3 times per week for 6–8 weeks. Results showed very small trends toward reduced hand pain and neuropathic symptoms, but the effects were highly variable and not statistically significant (Table 3). Since CIPN typically presents with more severe symptoms in the lower extremities than the upper extremities, hands-only training may not address patients' primary symptoms concerns. As demonstrated in the balance and strength RCTs, exercise programs can feasibly target both upper and lower extremities. Future RCTs shoulder consider interventions that combine upper and lower extremity exercises and evaluate upper and lower extremity outcomes separately, as was done by Simsek and Demir (43).

### 3.3 Acupuncture

Traditional acupuncture involves the insertion of needles at specific points on the body to promote “energy flow” and facilitate healing for variety of conditions (82). Practitioners emphasize the importance of accurate needling, which is characterized by a “pulling” sensation where the practitioner feels increased needle resistance, and the patient may experience numbness, soreness, or heaviness at the site of insertion. After decades of biomedical research, acupuncture is now hypothesized to function as a neuromodulatory treatment. The tissue sites commonly targeted for needling are thought to be richly innervated, and needling at these sites appears to trigger the release of endogenous opioids, along with other modulators, to aid the downstream reprocessing of pain signals (82, 83). Additionally, electroacupuncture, which involves delivering electric currents through needles, has shown potential regenerative effects. For example, animal models of traumatic nerve injury demonstrate increased mRNA expression of neurotrophic factors in the spinal cord and dorsal root ganglia following electroacupuncture, suggesting its ability to promote nerve regeneration (84).

Compared to oral medications and home-based exercise therapies, it is important to note that acupuncture faces significant accessibility challenges. The limited availability of licensed acupuncturists and the lack of insurance coverage for sessions create barriers for many patients. Out-of-pocket costs, the need to take time off work, and the logistical complexities of scheduling sessions alongside ongoing cancer treatment can make acupuncture an impractical option for many patients.

#### 3.3.1 Needle only acupuncture

Five RCTs evaluated needle-only acupuncture compared to sham, usual care, usual neuropathy rehabilitation, or waitlist control arms (Table 4) (30–34). Sample sizes ranged from 20 to 120 participants. The D'Alessandro 2019 RCT (*N* = 21), with the shortest treatment duration, assessed 5 weeks of acupuncture (10 sessions) plus neuropathy rehabilitation vs. neuropathy rehabilitation alone (30). This study reported a minimal trend toward pain reduction by 5 weeks, but with wide CIs. It remains unclear whether this nonsignificant outcome was due to the short treatment duration or the small sample size. However, the larger Molassiotis 2019 study (*N* = 87) also observed only a minimal trend toward pain improvement after 8 weeks of acupuncture, though with narrower CIs. This suggests that the limited pain outcomes seen in both studies may be attributed more to treatment properties than sample size (33). While the Molassiotis 2019 study did not show significant pain benefits, it did report improvement in sensory neuropathy outcomes for patient receiving acupuncture compared to control (Table 4).

As treatment duration increased to 9–10 weeks, as evaluated in 3 other RCTs, greater pain benefits were observed, even when acupuncture sessions occurred less than twice per week (*N* = 20–120). Additional improvements were also seen in symptom burden and functional measures (31, 32, 34). Friedemann 2022 (*N* = 102) was the only study to report follow-up data, collected 4 weeks after the end of treatment. They noted significant improvements in pain and numbness and a nonsignificant improvement in tingling (31). Most of these acupuncture RCTs involved patients with CIPN after completing chemotherapy; however, Stringer 2022's sample (*N* = 120) included 58% of participants with ongoing chemotherapy (34). The overall positive findings across studies suggest that acupuncture may be beneficial during chemotherapy as well as after its completion. Future trials should consider stratified randomization to better represent both groups and clarify potential differences.

In summary, needle-only acupuncture appears to be more effective for pain and sensory outcomes after 9–10 weeks of at least weekly sessions, with potential benefits extending up to 4 weeks after intervention. We lack data on the long-term efficacy of acupuncture for CIPN patients, but based on a meta-analysis of acupuncture for other chronic pain conditions, benefits may potentially last up to a year or more (85).

#### 3.3.2 Electroacupunture

Three RCTs, reflecting results from 4 published studies, evaluated electroacupuncture compared to placebo pills, usual care, or non-invasive sham acupuncture for CIPN patients after chemotherapy (Table 4) (26–29). Rostock 2013 (*N* = 31) conducted the shortest trial, with 3 weeks of 7–9 sessions of electroacupuncture compared to placebo pills, reporting minimal changes neuropathy symptoms (29). The two remaining RCTs, Bao 2020a/Bao 2021 (*N* = 45) and Lu 2020 (*N* = 33), featured 8 weeks of electroacupuncture, with former involving at least weekly sessions and the latter at least twice weekly sessions (26–28). Both weekly and biweekly electroacupuncture resulted in similar improvements in pain outcomes compared to usual care outcomes (−1.56 MD vs. −1.70 MD, Table 4). The biweekly electroacupuncture trial showed slightly greater benefits neuropathic symptoms than the weekly electroacupuncture (7.5 MD vs. 4.17 MD, Table 4).

The Bao 2020a/Bao 2021 study also provided 4-week follow-up data, showing symptoms relief benefits diminished over time. When electroacupuncture was compared to non-invasive sham acupuncture, there was only a minimal trend toward improvements in pain and tingling. It is important to note that designing real vs. sham acupuncture trials poses significant methodological challenges, particularly with placebo effects, expectancy, and participant blinding (86). Consequently, it is difficult to determine whether the minimal findings in electroacupuncture vs. non-invasive sham reflect methodological issues or a true lack of efficacy. This uncertainty is underscored by the Huang 2021 study (*N* = 20), which demonstrated pain benefits with needle-only acupuncture compared to invasive shallow needling sham, highlighting potential differences in sham acupuncture methodologies.

To summarize, electroacupuncture appears to provide symptom benefits for CIPN after 8 weeks of either weekly or twice weekly treatment, though these benefits may begin to wane as early as 4 weeks after the intervention ends. Compared to needle-only acupuncture, electroacupuncture may achieve pain benefits slightly faster (8 weeks vs. 9 weeks), but both interventions seem to offer similar overall benefits by the end of their respective treatment periods. No electroacupuncture RCTs have assessed outcomes in CIPN patients undergoing chemotherapy, leaving this population unstudied. Electroacupuncture shares the same risks and contraindications as needle-only acupuncture (vasovagal response, bleeding, infection, etc.) as well as has its own intervention-specific risks. Electroacupuncture carries the additional risks of skin pigmentation, electrical burns, muscle spasms, and rarely, cardiac blockade (87).

### 3.4 Emerging treatments

Briefly, we will discuss some of the most promising treatment options for CIPN beyond the commonly studied interventions. Each RCT mentioned here was cited only once across all SRs published in 2023, meaning the majority of SRs did not include these trials in their synthesis. They are presented and compared altogether for the first time below.

Three RCTs investigated foot reflexology, an alternative medicine technique combining massage and non-invasive pressure stimulation of acupuncture points (acupressure), compared to usual care or waitlist control (49, 50, 53, 88). Kurt and Can 2018 (*N* = 60) found that 6 weeks of twice daily reflexology sessions performed by relatives significantly improved sensory outcomes (−11.61 MD, 95% CI: −21.30 to −1.92 on a 0–100 point scale) (49). A year later, Noh and Park 2019 (*N* = 63) tested whether less frequent *self*-performed reflexology sessions could yield similar benefits but found no difference after 6 weeks of thrice weekly sessions (50). The most recent study by Gholamzadeh et al. 2023 (*N* = 80) showed that nurse-led once-weekly reflexology over 4 weeks produced similar benefits to the Kurt and Can 2018 study (−8.0 MD, 95% CI: −14.05 to −1.95 on the same 0–100 point scale) (53). These small RCTs suggest that non-self-performed reflexology may offer short-term benefit for sensory symptoms. However, due to the lack of sham reflexology arms, these benefits may be attributable to placebo effects arising from increased provider attention. Therefore, while the results are somewhat promising, it is difficult to definitively conclude whether reflexology is actually beneficial.

Kim and Park 2021 (*N* = 58) published an RCT examining self-acupressure of the hands and feet vs. neuropathy education. They found that 3 weeks of thrice daily self-performed acupressure reduced pain interference and overall CIPN symptoms (51). Unlike the Noh and Park 2019 study, this suggest that self-administered manual therapy may be effective if performed more frequently, or that acupressure alone could be more effective than reflexology. Larger RCTs are needed to better assess the true effects of manual therapies such as reflexology and acupressure.

Another manual therapy RCT by Jung et al. 2023 (*N* = 51) studied ear seeding, a common form of auricular acupressure in which small plant seeds are taped to specific areas of the ear, for CIPN sensory symptoms compared to sham auricular acupressure (52). Although it may seem unconventional, auricular acupressure has some evidence of effectiveness for pain management, likely through inhibition of pain signaling pathways, possibly via vagal nerve stimulation as the auricular branch of the vagus nerve innervates the outer ear canal (89, 90). In their RCT, both arms underwent seed taping to different parts of the ear weekly for 3 weeks, with seeds left in place for 5 days of each week. Results showed that compare to sham (seeds taped to ear lobe), patients who received proper auricular acupressure (seeds taped to mostly outer ear canal) experienced a large reduction in pain and a minor reduction in sensory symptoms by end of treatment (−2.50 MD, 95% CI: −3.29 to −1.71 on a 0–10 scale for pain; −1.94 MD, 95% CI: −3.35 to −0.52 on a 0–100 scale for sensory outcomes). At the 4-week follow-up, patients continued to experience pain relief, though the benefits were less pronounced than at the end of treatment. Sensory outcomes at follow-up were no longer significant. Further RCTs are needed to corroborate these findings and to explore whether vagus nerve stimulation may be the primary mechanism behind these effects.

## 4 Discussion

Based on an effect size analysis approach, we conclude that that duloxetine and venlafaxine are likely beneficial for patients with CIPN after chemotherapy completion, though benefits may take 4–5 weeks to appear, and the duration of these benefits remains unknown. We argue that venlafaxine may be a reasonable alternative to duloxetine when appropriate. Gabapentin is unlikely to benefit, while pregabalin and TCAs may have small but likely insignificant benefits. Needle-only acupuncture or electroacupuncture could also be a treatment option for patients with CIPN after chemotherapy completion, but patient may require 8–10 weeks of weekly or biweekly treatment for benefits to appear. The duration of benefits is similarly unclear, and accessibility may be limited due to costs and logistical challenges. For patients with CIPN who are actively undergoing chemotherapy, there is limited data on the efficacy of SNRIs or acupuncture, but these options may still be worth considering as part of the treatment plan.

For patients with CIPN who are undergoing chemotherapy, regular home-based balance and strength training may reduce CIPN symptoms and functional interference, but likely requires consistent exercise for 10 or more weeks. It is unstudied whether exercise benefits CIPN symptoms in patients after chemotherapy treatment, but it remains a reasonable option to try.

We have highlighted several areas for improvement for future SRs and clinical trials in the field of CIPN treatment. Through an umbrella review approach, we demonstrate that SRs are often less systematic than expected. None of the 19 CIPN treatment SRs published in 2023 captured all relevant RCTs within their search timeframe. However, by cross-referencing SRs, we identified 41 RCTs across 42 publications, illustrating the feasibility of an umbrella review approach to uncover relevant trials.

Furthermore, many SRs exhibited methodological concerns that limit the quality and interpretability of their findings. The majority of SRs included comparative effectiveness trials (79%) and combined analyses of both preventative and treatment trials (63%). In the context of CIPN, where no widely accepted standard of care exists, comparative trials can sometimes obscure rather than clarify treatment effects. For example, if one experimental treatment is beneficial while another is harmful, their direct comparison may exaggerate the perceived benefit of the effective arm without providing clear evidence of its absolute efficacy.

Additionally, the inclusion of both prevention and treatment trials in a single analysis introduces significant heterogeneity. Prevention trials typically focus on the incidence of CIPN as the primary outcome, with symptom severity as a secondary measure. In contrast, treatment trials assess symptom reduction in patients with established CIPN. Comparing secondary outcomes from prevention trials with primary outcomes from treatment trials risks introducing bias, as these subgroup analyses are no longer randomized and may not be directly comparable.

While ASCO guidelines have recommended duloxetine, CIPN management remains heterogeneous, and no universally accepted standard of care exists (91, 92). Duloxetine is the only pharmacologic agent supported by two randomized trials, yet adherence to this recommendation is inconsistent, and many patients do not tolerate or benefit from the medication. Furthermore, as noted in recent reviews, clinician opinions continue to reflect a lack of a broadly endorsed, standardized approach to CIPN assessment and management (91–93).

To improve methodological rigor, future SRs evaluating CIPN treatment efficacy should focus on trials with appropriate control arms and on patient populations with pre-existing CIPN. SRs assessing comparative effectiveness should build on these efficacy-focused reviews, incorporating only well-established interventions as comparators (e.g., once SNRIs are consistently shown to be effective, an SR could examine comparative trials involving SNRIs and other treatments). This approach would help ensure that comparative analyses are based on robust evidence and minimize the risk of misleading conclusions due to methodological inconsistencies.

We have also discussed multiple opportunities for refining methods and reporting in CIPN treatment trials throughout this article. First, trials should report post-intervention or change-from baseline score for every outcome measure mentioned in the methods section, along with metrics reflecting data variance (CIs, standard deviation, or standard error). This endures data transparency and allows for readers to properly evaluate outcomes independently, rather than relying solely on authors’ interpretations.

Second, selected outcome measures should ideally reflect clinically relevant constructs that are easily translatable for patient counseling, such as the percent of individuals achieving a 50% or greater pain reduction. While quality-of-life outcome measures and other ordinal measures may show measurable improvement post-intervention, they cover broad domains that are difficult to translate into meaningful, dichotomized rates for calculating measures such as RR and NNT. RR and NNT calculations require dichotomous data to construct a 2 × 2 contingency table. If studies report continuous or ordinal outcome measures as solely summary statistics, RR and NNT cannot be directly computed unless two conditions are met: (1) access to individual patient-level data and (2) a standardized, transparent method for dichotomizing outcomes. Consequently, we were unable to derive RR and NNT for many of the non-pharmacologic studies.

Finally, effect size analysis plays a crucial role in evaluating the true magnitude of treatment effects, beyond the limitations of statistical significance alone. We emphasize that effect sizes and their CIs offer a more meaningful way to interpret clinical relevance, especially in studies where small sample sizes or high variability may obscure significant *p*-values.

Regarding limitations, this is a non-comprehensive focused analysis of the CIPN treatment interventions that have the most RCT evidence. This does mean that promising non-randomized or single arm trials, such as with topical agents like capsaicin, were outside the scope of this study. These studies are more susceptible to confounding factors, such as placebo effects, regression to the mean, or differences in patient characteristics, making it unclear whether these findings translate into true clinical benefits. Nonetheless, early evidence for therapies like topical treatments warrant further investigation in well-designed RCTs.

Additionally, we restricted this umbrella review to SRs published in 2023 to ensure a contemporary synthesis of available evidence. The high volume of SRs published in a single year (*n* = 19) suggested a potential oversaturation, making this timeframe an optimal choice for achieving reasonable saturation while still capturing a broad range of relevant RCTs. While it is possible that some RCTs were not included in any of the 19 SRs, our approach did identify multiple instances where RCTs on similar topics were analyzed separately in different SRs rather than collectively. This underscores the added value of our umbrella review in providing a more integrated and comprehensive evaluation of CIPN treatment trials.

Lastly, there was significant heterogeneity in cancer types, stages, and chemotherapy regimens across the included studies. While this variability limits the precision of findings for any single patient population within CIPN, it also reflects the inherent complexity of real-world clinical practice, where patients often undergo diverse treatment protocols. Our approach acknowledges and transparently navigates this heterogeneity, allowing for meaningful comparisons across treatment modalities. Although not specific to any one cancer type or demographic, the effect sizes synthesized here provide a structured appraisal of commonly studied CIPN interventions, offering practical insights despite the variability.

## 5 Conclusion

This focused umbrella review was conducted to identify and synthesize the outcome evidence from recently published SRs on the most common interventions for established CIPN. Despite a wealth of published research, cancer survivors have limited options for successfully managing CIPN. Since the release of the 2020 ASCO guidelines for CIPN management, several additional RCTs and SRs have been published. Using an umbrella review approach, we identified a total of 41 RCTs identified across 19 SRs published in 2023. Effect size analysis of the most common interventions reveal that SNRIs, home-based balance and strength training, and acupuncture can be beneficial in certain circumstances for patients with CIPN. Emerging data on manual therapies, such as massage and acupressure, also show promise, but further trials are needed to clarify key questions. The focused umbrella review approach, which involved identifying SRs and then compiling relevant RCTs, allowed us to aggregate more RCTs than have been analyzed together in any one SR, demonstrating the feasibility and utility of this approach for data synthesis. Finally, we highlighted several opportunities for improving both SRs and RCTs, focusing on addressing current limitations in the CIPN literature, enhancing methodological rigor, and refining the way studies are designed and reported to better inform future research.

## Data Availability

The raw data supporting the conclusions of this article will be made available by the authors, without undue reservation. Requests to access the raw data should be directed to Alice L. Ye at alye@mdanderson.org.
