# Supplementary material for: Beyond p-values: a cross-sectional umbrella review of chemotherapy-induced peripheral neuropathy treatments
Source: Front Pain Res (Lausanne). 2025 Mar 19;6:1564662. doi: 10.3389/fpain.2025.1564662 (PMC11961875; doi:10.3389/fpain.2025.1564662)
Supplement: Supplementary file 1 [file Table1.docx]

**Search Strategy**

Database(s): Ovid MEDLINE(R) ALL 
Search Strategy:

| **#** | **Searches** |
| --- | --- |
| 1 | peripheral nervous system diseases/ or polyneuropathies/ or small fiber neuropathy/ |
| 2 | (peripheral adj3 (neuropath* or polyneuropath* or neurotoxic*)).ti,ab,kf. |
| 3 | CIPN.ti,ab,kf. |
| 4 | or/1-3 |
| 5 | (animals not (humans and animals)).sh. |
| 6 | 4 not 5 |
| 7 | (animal* or mice or mouse or murine or rodent or rat or rats or rodent or cells or "in vitro" or "cell line").ti. |
| 8 | 6 not 7 |
| 9 | limit 8 to english language |
| 10 | exp Neoplasms/ |
| 11 | (cancer* or carcinom* or neoplas* or chemotherapy or myeloma* or leuk?emia* or lymphoma*).ti,ab,kf. |
| 12 | 10 or 11 |
| 13 | 9 and 12 |
| 14 | systematic review*.ti,pt. or "cochrane database of systematic reviews".jn. or meta-analysis as topic/ or Meta-Analysis.pt. or (meta-analy* or metaanaly*).ti. |
| 15 | 13 and 14 |
| 16 | case reports.pt. or case report*.ti. or case series.ti. or (case or cases).ti. |
| 17 | 15 not 16 |

Database(s): Embase Classic+Embase
Search Strategy:

| **#** | **Searches** |
| --- | --- |
| 1 | *peripheral neuropathy/ or small fiber neuropathy/ |
| 2 | (peripheral adj3 (neuropath* or polyneuropath* or neurotoxic*)).ti,ab,kf. |
| 3 | CIPN.ti,ab,kf. |
| 4 | or/1-3 |
| 5 | exp malignant neoplasm/ |
| 6 | (cancer* or carcinom* or neoplas* or myeloma* or leuk?emia* or lymphoma* or chemotherapy).ti,ab,kf. |
| 7 | 5 or 6 |
| 8 | 4 and 7 |
| 9 | chemotherapy-induced peripheral neuropathy/ |
| 10 | 8 or 9 |
| 11 | limit 10 to english language |
| 12 | (Nonhuman/ or ANIMAL/ or Animal Experiment/) not Human/ |
| 13 | 11 not 12 |
| 14 | (animal* or mice or mouse or murine or rodent or rat or rats or rodent or cells or "in vitro" or "cell line").ti. |
| 15 | 13 not 14 |
| 16 | exp Cochrane Library/ or exp "systematic review (topic)"/ or exp "systematic review"/ or meta analysis/ or (systematic review* or meta-analy* or metaanaly*).ti. |
| 17 | 8 and 16 |
| 18 | case report/ or case report*.ti. or case series.ti. or (case or cases).ti. |
| 19 | 17 not 18 |
| 20 | conference abstract.pt. |
| 21 | 19 not 20 |
